# Supplementary material for: TastepepAI: An artificial intelligence platform for taste peptide de novo design
Source: PLoS Comput Biol. 2025 Oct 16;21(10):e1013602. doi: 10.1371/journal.pcbi.1013602 (PMC12543283; doi:10.1371/journal.pcbi.1013602)
Supplement: S1 File — (ZIP) [file pcbi.1013602.s006.zip › Supplementary Data 2/TaPep1 HPLC.doc]

**HPLC REPORT**

| Sample: | FCL-NJP93301 TaPep1 AL-7 | | | Analyzed date: | 2024-9-25 |
| --- | --- | --- | --- | --- | --- |
| Analyst: | WJ | | | Reconstitution: | 100%H_2_O |
| Lot. No.: | NJP93301-1172214 | | | | |
| Column: | 4.6×250mm,Venusil MP C18-5 | | | | |
| Solvent A | A: 0.1% Trifluoroacetic Acid in 100% Acetonitrile | | | | |
| Solvent B | B: 0.1% Trifluoroacetic Acid in 100% Water | | | | |
| Gradient: |  | A | B |  | |
|  | 0.0min | 5% | 95% |  | |
|  | 25.0min | 30% | 70% |  | |
|  | 25.1min | 100% | 0% |  | |
|  | 30.0min | Stop | |  | |
| Volume: | 5μl | | |  | |
| Wavelength: | 220nm | | |  | |
| Flow rate: | 1.0ml/min | | |  | |

─────────────────────────────────────────────────────────────

Rank	Time	Conc. Area	Height

─────────────────────────────────────────────────────────────

1	6.832	0.2287	16819	2343

2	8.477	96.3332	7083703	491736

3	8.830	1.1549	84925	22586

4	9.066	1.8223	133997	12217

5	9.988	0.1333	9799	1315

6	10.437	0.3276	24092	2990

────────────────────────────────────────────────────────────

Total	100	7353335	533187
